# Application of the Six-Step Standard Communication Process in the Communication Training for Newly Recruited Nurses in Cancer Specialist Hospitals

**DOI:** 10.3389/fsurg.2022.842716

**Published:** 2022-02-08

**Authors:** Jingzhi Geng, Miao Liu, Huanhuan Zhang, Jian Gao, Li Wang, Yu Zhang, Fengyan Ma, Yan Liu

**Affiliations:** ^1^Department of Gynecologic Oncology, National Cancer Center/National Clinical Research Center for Cancer/Cancer Hospital, Chinese Academy of Medical Sciences Peking Union Medical College, Beijing, China; ^2^Department of Thoracic Surgery, National Cancer Center/National Clinical Research Center for Cancer/Cancer Hospital, Chinese Academy of Medical Sciences Peking Union Medical College, Beijing, China; ^3^Department of Radiation Therapy, National Cancer Center/National Clinical Research Center for Cancer/Cancer Hospital, Chinese Academy of Medical Sciences Peking Union Medical College, Beijing, China; ^4^Department of VIP Medical Services, National Cancer Center/National Clinical Research Center for Cancer/Cancer Hospital, Chinese Academy of Medical Sciences Peking Union Medical College, Beijing, China

**Keywords:** six-step standard communication process, cancer specialist hospital, new nurses, clinical communication skills, self-efficacy

## Abstract

**Purpose:**

Discuss the application effect of the six-step standard communication process in the communication ability training of newly recruited nurses.

**Methods:**

This is a before and after control study. The control group included 45 newly recruited nurses in our hospital in 2019, and the observation group included 40 newly recruited nurses in our hospital in 2020. The control group completed the training according to the existing communication training program, and the observation group implemented a training program based on the “six-step standard communication process” on the basis of the existing communication training. The training period was 12 months. The training effect of the two groups of new nurses was compared.

**Results:**

After training, the total scores of clinical communication skills of the new nurses in the control group and observation group were 252.56 ± 24.950 and 268.05 ± 19.335 points, respectively; the total scores of communication behavior were 39.00 ± 4.676 and 48.08 ± 2.515 points, respectively; the total scores of general self-efficacy were 26.89 ± 3.017 and 31.25 ± 5.027 points, respectively; the satisfaction scores of communication training were 17.56 ± 2.018 and 19.45 ± 0.986 points, respectively, and the differences were statistically significant (*P* < 0.05).

**Conclusion:**

The implementation of a training program based on the “six-step standard communication process” can effectively improve the clinical communication skills and self-efficacy of newly recruited nurses, and can be promoted and applied to the communication training of newly recruited nurses.

## Introduction

A specialized tumor hospital is a technically sophisticated and specialized hospital with the prevention and treatment of tumor diseases as its main business scope. Most of the patients it receives are special groups who are facing death threats and bear huge psychological pressure, so the oncology specialist hospital needs to show more humanistic care while providing them with superb medical technology. Nursing-patient communication is a key link in expressing humanistic care in the operation of tumor specialized hospitals. It plays a very important role in increasing patient satisfaction and improving clinical services, and most of the complaints of patients are related to poor doctor-patient or nurse-patient communication (1, 2). It has been reported that the problem of insufficient communication skills among newly recruited nurses is particularly prominent, and the nurse-patient communication skills they have mastered cannot meet their job needs (3, 4). And in the existing training, the communication training of newly recruited nurses is more inclined to theoretical learning and experience introduction, and there are problems such as low practicability and insignificant effect, so it cannot meet the special nursing needs of tumor hospitals for tumor diseases (5). In 2016, my country required communication ability training for new nurses in the “Training Outline for Newly Enrolled Nurses (Trial)” (6). The six-step standard communication process is formed by domestic scholars through the Chineseization and revision of the CICARE communication model, namely “connect-introduce-communicate-ask-respond-exit,” it streamlines and standardizes every communication between nurses and patients, which helps to rapidly improve the professionalism and communication skills of nurses, thereby providing better nursing support for patients. It is an effective communication method to build nurse-patient trust and promote nurse-patient harmony (7). This study intends to combine the nursing characteristics and clinical needs of cancer patients to construct a communication skills training program for newly recruited nurses based on the “six-step standard communication process,” and to explore its application effects. The report is as follows.

## Materials and Methods

### Research Object

Nurses who had been employed in our hospital for <1 year in 2019 and 2020. The new nurses were divided into a control group and an observation group according to their entry time. The control group included 45 newly recruited nurses in our hospital in 2019, and the observation group included 40 newly recruited nurses in our hospital in 2020. Inclusion criteria: ① Those who had not received the six-step standard communication process training; ② Those who had passed the nurse qualification examination; ③ Those who had a college degree or above. Exclusion criteria: ① Those who could not participate in the whole training; ② Those who had work experience; ③ Those who worked in the operating room, ICU, and auxiliary examination departments after entering the job.

### Training Program

Nurses in the control group received communication training according to the existing program. This included theoretical lectures on communication-related knowledge, simulation training on communication, communication case analysis and discussion, communication practice under the supervision of a specialist during clinical work, participation in clinical department work, ward rounds and business learning, learning the communication style and skills of teaching teachers, and timely guidance from teaching teachers on communication problems arising from new nurses.

The observation team implemented a training program based on the “six-step standard communication process” on the basis of the control group. Specifically, this includes: ① Establishing a training team. The team leader was the associate director for teaching in the nursing department, and the team consisted of six members. They included head nurses, teaching teachers, quality control nurses, the titles were all nurses in charge and above. ② Developing a six-step standard communication flow chart and publicity posters and posting them in the departments and wards where the observation group nurses were located. The main contents included: connect (polite contact: using the preferred term of address when approaching patients and families), introduce (enthusiastic introduction: introducing yourself and explaining duties), communicate (explain in detail: what would be done to communicate with the patient, how long it would take, and how it would affect the patient), ask (careful inquiry: obtaining consent before performing the procedure and asking the patient's needs), respond (patiently answer: patiently answering questions from patients or families), exit (leave politely: politely explaining what needed to be done next, saying goodbye politely). ③ Production of training materials. The aforementioned flowchart would be made into a manual and distributed to new nurses; a video of scenario-based communication simulation would be filmed for new nurses to learn. ④ Establishing a “communication typical case library.” Each department summarizing clinical nursing scenarios according to the characteristics of diseases, and the training team selecting typical cases and using the “six-step standard communication process” to create a “communication typical case library,” which was divided into common nursing situations such as receiving consultation, intravenous infusion, health education and psychological guidance. ⑤ Training implementation. Divided into 3 phases. Phase 1 was the theoretical lecture phase. Conducting lectures on related knowledge of the “six-step standard communication process,” mainly training its connotation, definition, communication skills knowledge and specific applications. At the same time, citing cases and watching scenario simulation videos, inspiring members to ask questions, and solve difficulties and doubts in communication in time. Phase 2 was the scenario training stage. Using standardized patients, new nurses can experience different scenarios and feel the communication skills in special situations, so that theory and practice can be integrated to strengthen in-depth understanding of the communication process. Phase 3 was the clinical practice and consolidation stage. Implementing the “six-step standard communication process” in clinical work, and making a nursing summary of the problems encountered in the actual operation, and the nurses shared actual work cases and exchanged discussions with each other.

### Research Tools

General information questionnaire: It was designed by the researchers themselves, including the gender, age, marital status, education background of the research object, whether it was an only child, the attitude of choosing a nursing major, and whether they had participated in communication skills training, etc.

Nurses' clinic communication competence scale (NCCCS): Including 6 dimensions, a total of 58 items. The KMO of the scale was 0.972, the overall Cronbach's α coefficient was 0.978, and the Cronbach's α coefficient of each dimension was between 0.873 and 0.954. Using the Likert 5-level scoring method, each item was assigned a value of 1 to 5 points from “very poor” to “very good,” and the score ranged from 58 to 290 points. All items were scored positively, the higher the score, the better the communication skills.

Clinical communication behavior scale: The content included 10 entries. A Likert 5-point scale (1=very poor, 5=very good) was used. each item was assigned a score of 1 to 5 from very poor to very good, with a total score of 10 to 50. The higher the score, the better the communication ability.

General self-efficacy scale (GSES): The total Cronbach's α coefficient of the scale was 0.87, and the test-retest reliability was 0.83 (8). The Chinese version of GSES had a total of 10 entries, all of which were forward entries. The Likert 4-level scoring method was used to score 1–4 points from “completely incorrect” to “completely correct,” with a total score of 10–40 points. The higher the score, the stronger the nurse's sense of self-efficacy. The self-efficacy score of the international norm was (28.6 ± 4.0) points.

Nurse communication training satisfaction questionnaire: The questionnaire consisted of 5 items and was scored on a 4-point scale from 1 to 4, ranging from “strongly disagree” to “strongly agree,” with higher scores indicating higher satisfaction. The questionnaire was reviewed by experts and had good reliability and validity, with a Cronbach's S coefficient of 0.85 and a retest reliability of 0.95.

### Evaluation Method

After 12 months of new nurses' training, a questionnaire was distributed by the research team to investigate the new nurses' clinical communication ability, self-efficacy and satisfaction with communication training in 2019 and 2020, respectively, while the new nurses' communication behaviors in clinical nursing were rated by a dedicated person.

### Statistical Analysis

Count data were expressed as number of cases and composition ratio (%), and the chi-square test was used for comparison between groups; normally distributed measures were expressed as mean ± standard deviation, and the independent samples *t*-test was used for comparison between groups. Differences were indicated as statistically significant at *P* < 0.05.

## Results

### General Information of New Nurses

The differences in general information between the new nurses in the control and observation groups were not statistically significant (*P* > 0.05) and were comparable. As seen in Table 1.

**Table 1:** General information of new nurses.

**Items**	**Control group (***n*** = 45)**	**Observation group (***n*** = 40)**	***t*** **/χ^2^ value**	***P*** **-value**
**Gender** Male Female	0 (0.00%) 45 (100.00%)	0 (0.00%) 40 (100.00%)		
**Average age**	23.31 ± 1.08	22.80 ± 0.97	2.279	0.025
**Only child or not** Yes No	19 (42.22%) 26 (57.78%)	11 (27.50%) 29 (72.50%)	2.010	0.156
**Residence** Urban Rural	18 (40.00%) 27 (60.00%)	21 (52.50%) 19 (47.50%)	1.333	0.248
**Education level** College Bachelor or above	20 (44.44%) 25 (55.56%)	16 (40.00%) 24 (60.00%)	0.171	0.679
**Personality type** Outgoing Introverted	31 (68.9%) 14 (31.1%)	25 (62.50%) 15 (37.50%)	0.385	0.535
**Have received communication training before entering the job**				
Yes	37 (82.22%)	8 (17.78%)	0.455	0.500
No	35 (87.50%)	5 (12.50%)		

### NCCCS Score of New Nurses

**Figure 1:**
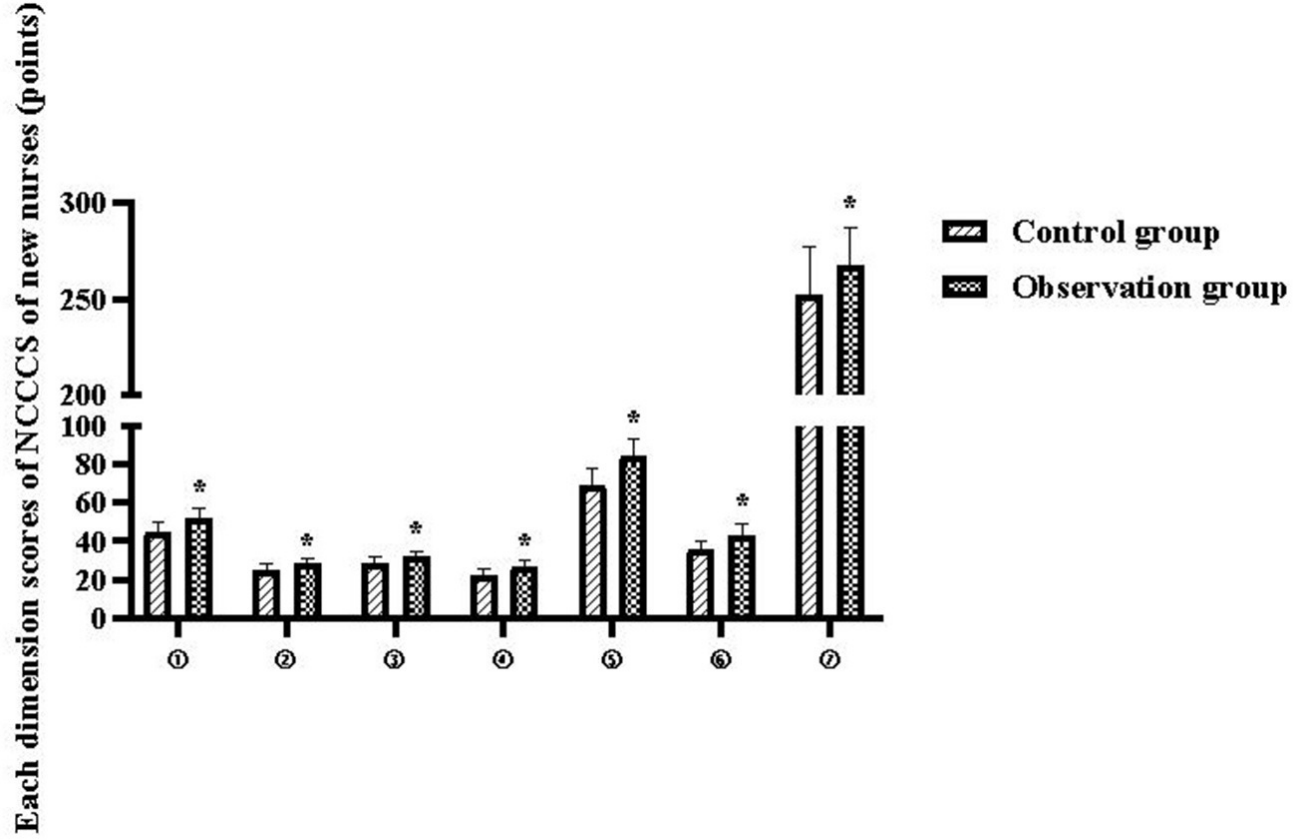
Each dimension scores of NCCCS of new nurses. Compared with the control group, *P < 0.05.

The total NCCCS scores of new nurses in the control and observation groups were 252.56 ± 24.95 and 268.05 ± 19.34 points, respectively (*P* < 0.05), and the scores of each dimension are shown in Table 2 and Figure 1.

**Table 2:** NCCCS scale of new nurses.

**Serial number**	**Number of entries**	**Dimension**
①	11	Basic verbal communication skills
②	6	Team communication skills
③	7	Basic non-verbal communication skills
④	6	Emotional support capabilities
⑤	19	Communication skills in difficult situations
⑥	9	Emotional perception ability
⑦	58	Communication total items

### Clinical Communication Behavior Score of New Nurses

**Figure 2:**
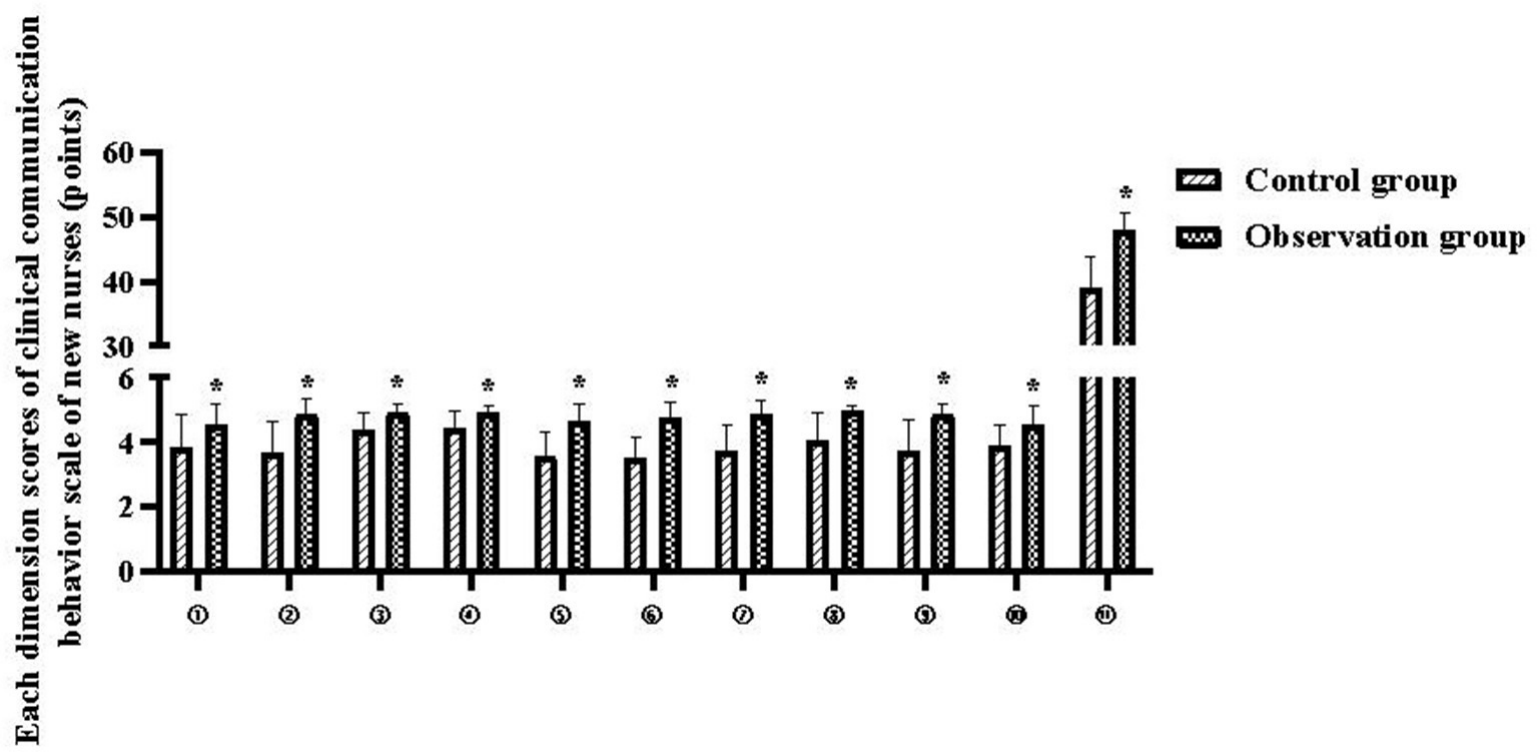
Each dimension scores of clinical communication behavior scale of new nurses. Compared with the control group, *P < 0.05.

The total clinical communication behavior scores of new nurses in the control and observation groups were 39.00 ± 4.68 and 48.08 ± 2.52 points, respectively (*P* < 0.05), and the scores of each dimension are shown in Table 3 and Figure 2.

**Table 3:** Clinical communication behavior scale of new nurses.

**Serial number**	**Item**
①	Knock before entering the door
②	Introduce yourself before communicating
③	Inform and explain when communicating
④	Gaze when communicating
⑤	Pay attention to the patient's reaction when communicating
⑥	Ask for patients' opinions when communicating
⑦	Answer questions from patients during communication
⑧	Leave politely
⑨	Put the stool away when leaving and close the door gently
⑩	Overall evaluation
⑪	Total score

### GSES Score of New Nurses

**Figure 3:**
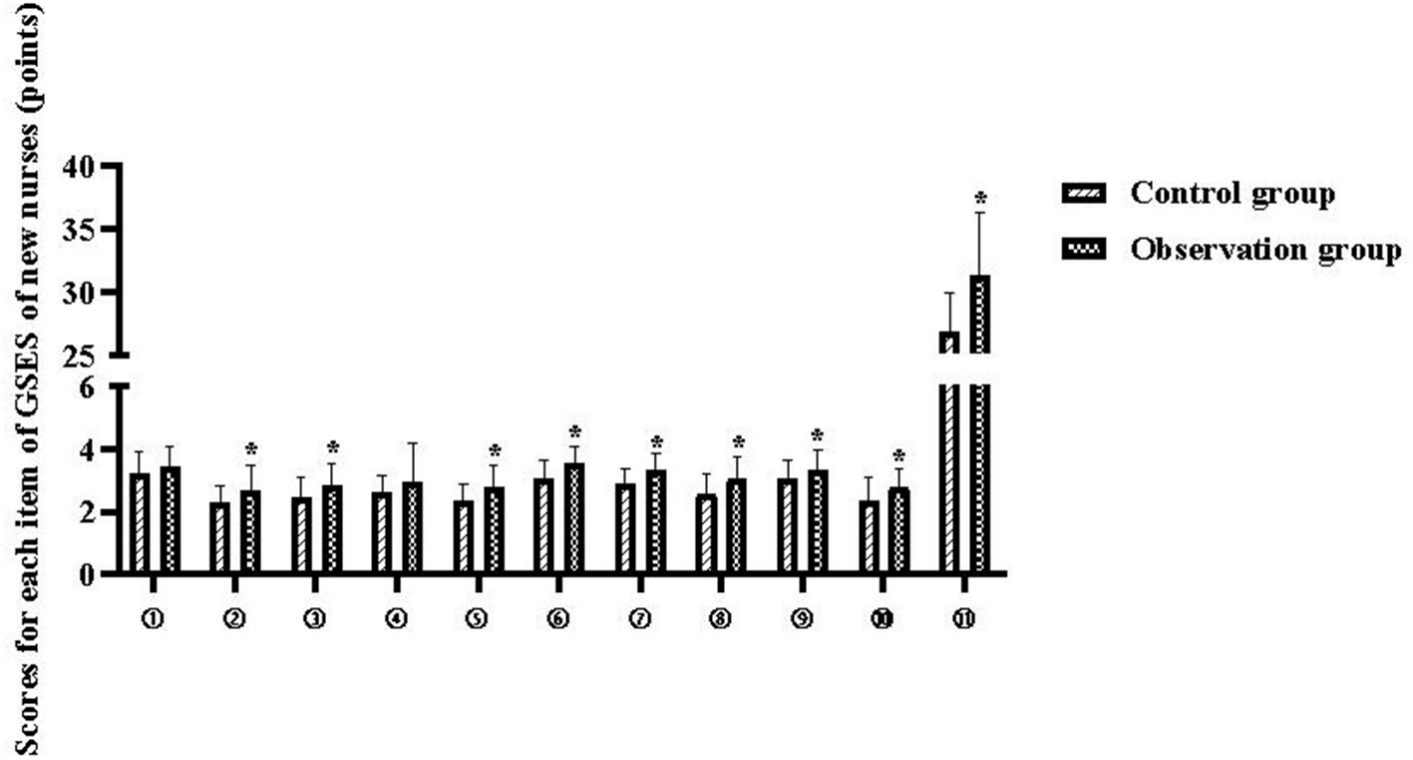
Each item scores of GSES of new nurses. Compared with the control group, *P < 0.05.

The total GSES scores of new nurses in the control and observation groups were 26.89 ± 3.02 and 31.25 ± 5.03 points, respectively (*P* < 0.05), and the scores of each items are shown in Table 4 and Figure 3.

**Table 4:** GSES scale of new nurses.

**Serial number**	**Item**
①	If I do my best, I can always solve the problem
②	Even if others oppose me, I still have the means to get what I want
③	It is easy for me to stick to my ideals and reach my goals
④	I am confident that I can deal effectively with anything that comes up unexpectedly
⑤	With my talent, I'm sure I can handle the unexpected
⑥	If I put in the necessary effort, I will be able to solve most of the puzzles
⑦	I can face difficulties calmly because I trust my ability to deal with problems
⑧	When faced with a difficult problem, I can usually find several solutions
⑨	When there is trouble, I can usually think of some ways to deal with it
⑩	No matter what happens to me, I can handle it
⑪	Total score

### Communication Training Satisfaction Score of New Nurses

**Figure 4:**
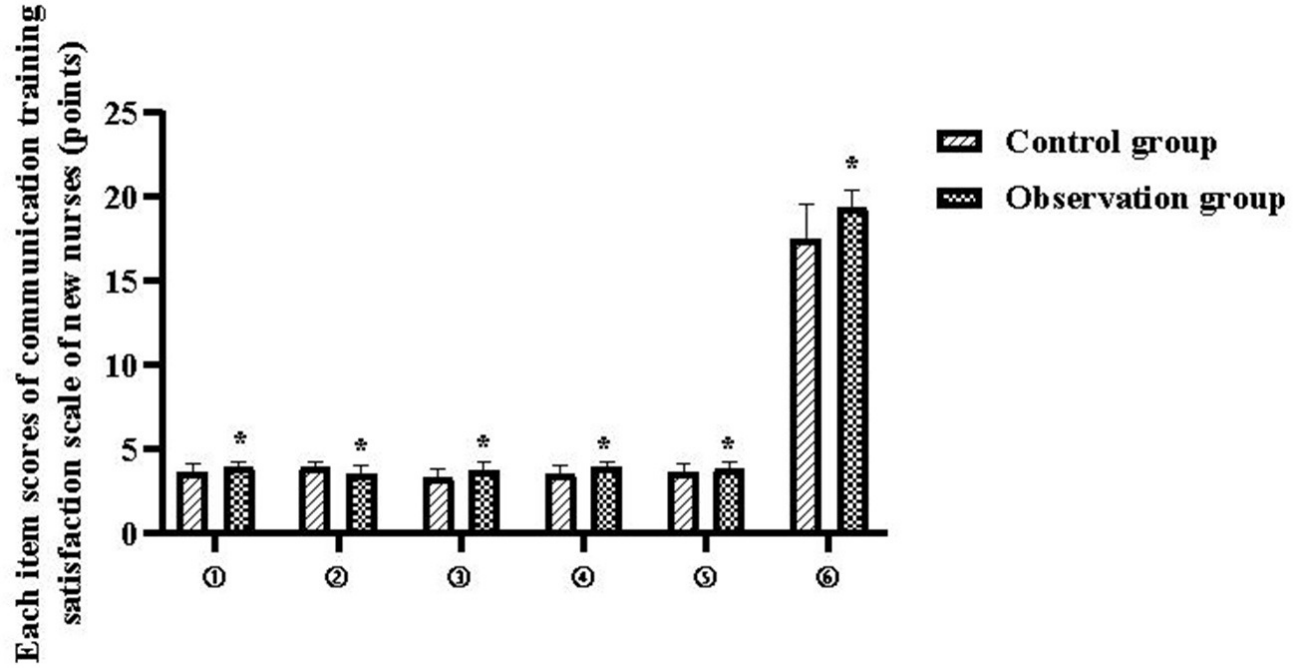
Each item scores of communication training satisfaction scale of new nurses. Compared with the control group, *P < 0.05.

The total communication training satisfaction scores of new nurses in the control and observation groups were 17.56 ± 2.02 and 19.45 ± 0.99 points, respectively (*P* < 0.05), and the scores of each item are shown in Table 5 and Figure 4.

**Table 5:** Communication training satisfaction scale of new nurses.

**Serial number**	**Item**
①	I was generally very satisfied with the way the communication course was conducted
②	I think the course content is presented in an easy-to-understand way, easy to imitate and remember
③	I have put the communication skills I have learned to good use in my daily work
④	I am more confident in my work and daily communication
⑤	The use of communication skills has made my interpersonal relationships more harmonious
⑥	Total score

## Discussion

New nurses lack communication skills and experience, and often do not know how to communicate properly when in contact with patients. And in view of the particularity of tumor diseases, patients and their families have shown more nursing needs for medical staff, which puts forward stricter nursing communication requirements for newly recruited nurses in cancer hospitals. This study applied the “six-step standard communication process” to the training of newly recruited nurses' communication skills, and the results showed that:

After training, in clinical communication ability and communication behavior, the total score and each item score of the observation group were significantly higher than those of the control group (*P* < 0.05). This suggests that training based on the “six-step standard communication process” can effectively improve the clinical communication skills of newly recruited nurses in cancer specialist hospitals and promote the norms of communication behavior. The cultivation of nurse-patient communication skills of newly recruited nurses is an important part of nursing human resource management, and it is also a necessary measure to meet the needs of patients and ensure the quality of clinical care. The “six-step standard communication process” is a training method proposed by the University of California, Los Angeles General Hospital, which is divided into six processes of “connect-introduce-communicate-ask-respond-exit.” Its content covers standard phrases and procedures for communication with patients, and its core contains communication details such as respect, courtesy, communication, civility, listening and smiling, which can provide a practical template for new nurses' clinical communication and can better transition communication skills from theory to practice. And in the form of communication, it goes from shallow to deep, from polite conversation to declarative communication, and up to emotional communication, so that the theory of nursing communication becomes easy to understand and operable from complicated, which is convenient to correct irrational understanding, improve communication ability and solve patients' practical problems. The application of the six-step standard communication process in this study, through theoretical lectures, posters, the production of training materials, and the filming of scenario-based communication videos, can enable new nurses to quickly grasp the specific definition, connotation and application of the six-step standard communication process, and actively create an atmosphere of nurse-patient communication using the six-step standard communication process; At the same time, each department summarizes clinical nursing scenarios according to disease characteristics, and the training team selects typical cases and uses the “six-step standard communication process” to create a “communication typical case library,” which can provide new nurses with rich, typical and realistic learning cases. The above nursing training process is interlocked and step-by-step, which can guide new nurses to have friendly contact with patients and strengthen communication, so that nurses can know the specific communication methods and contents each time they serve patients, which is an important way to realize the rapid transformation of humanistic theoretical knowledge into practical application, rapidly improve the clinical communication ability of new nurses, create a harmonious treatment environment, and promote the standardization and homogenization of nursing services (9).

After training, the total score of general self-efficacy and communication training satisfaction scores of the observation group were higher than those of the control group, and the total score of self-efficacy of the observation group was higher than the international norm value (28.6 ± 4.0) points (*P* < 0.05). This suggests that training based on the “six-step standard communication process” can enhance the self-efficacy and job confidence of newly recruited nurses in cancer specialist hospitals, and increase satisfaction with communication training. Self-efficacy is a concept proposed by the American psychologist Bandura in 1977. It refers to people's confidence or belief in their ability to achieve behavioral goals in a specific field (10). Nurses' self-efficacy is positively correlated with their nursing behaviors (11–13). Nurses with a high sense of self-efficacy can effectively exercise self-control, regulate their own behaviors, and promote professional behaviors in nursing practice. By improving their sense of self-efficacy, they can promote nurses to increase their work rate and work quality, and reduce their tendency to leave, and ultimately increase quality of nursing service. In addition, nurses with stronger communication skills can also positively influence the overall sense of experience and nursing satisfaction of cancer patients and have the effect of improving the overall health status of patients, however, new oncology nurses rarely receive formal nursing communication instruction during clinical learning, and with insufficient theoretical knowledge and lack of practical experience, they often fall into a flustered and passive situation when communicating with patients, and therefore have a low sense of self-efficacy, which is not conducive to the cultivation of work confidence and the establishment of a harmonious nurse-patient relationship (14–16). This study implements a six-step standard communication process that integrates theory and practice. Compared with the previous communication training process, the content is not only easier to understand and easy for new nurses to imitate and remember, but also helps to cultivate new nurses' critical thinking and exercise their ability to analyze and solve practical problems. For example, the application of standardized patient scenarios during training allows new nurses to experience different scenarios and perceive nursing communication skills in special situations, which helps them to have a deeper understanding of communication processes and skills and be able to apply them flexibly in conjunction with actual clinical needs.

As a result, the new nurses' overall clinical competence improved significantly, and their self-efficacy and satisfaction with the communication training increased accordingly.

The results of this study also showed that in GSES, the scores of entries and were higher in the observation group than in the control group, but there was no statistical difference (*P* > 0.05). The reason may be that the training subjects in this study were new nurses in oncology hospitals, and although a “communication typical case library” was established and training on “communication in difficult scenarios” was conducted, oncology nursing is faced with more complex diseases and patients, which has higher demands on nursing staff (17, 18). Therefore, new nurses still do not feel confident that they can “always solve problems” and “deal effectively with anything that comes up” due to their lack of oncology nursing knowledge and clinical experience.

Cancer treatment is a long and complicated process. In this process, patients and their families suffer from multiple burdens from mental, physical and economic aspects, and nurses are faced with various demands from patients, so they must treat each patient with “love, patience, care, compassion and responsibility,” continuously improve service quality and service attitude, and really make our nursing work to the heart of patients (19, 20). The six-step standard communication process of this study practiced the “people-oriented” nursing concept throughout the entire process. It focuses on the needs of patients, pays attention to evaluating and meeting the needs of patients, fully embodies the nursing philosophy of humanistic care, and helps new nurses to win the satisfaction of patients. The increase in positive reviews received by new nurses in nursing work also contributes to the improvement of self-efficacy and job satisfaction (21, 22).

## Conclusion

Training based on the “six-step standard communication process” can improve the communication skills and self-efficacy of new nurses in cancer specialist hospitals, enhance the confidence of new nurses in communication, and enable them to quickly master nurse-patient communication skills, and effectively apply them in clinical nursing work. It is worth promoting and applying in the communication training of newly recruited nurses. However, due to the short tracking time of this study, the long-term maintenance effect of this training model in the clinical communication of newly recruited nurses needs to be further tracked and refined.

## Data Availability Statement

The original contributions presented in the study are included in the article/Supplementary Material, further inquiries can be directed to the corresponding author.

## Ethics Statement

The studies involving human participants were reviewed and approved by the Ethics Committee of Chinese Academy of Medical Sciences Peking Union Medical College. The patients/participants provided their written informed consent to participate in this study.

## Author Contributions

JGe and ML are mainly responsible for the writing of the manuscript. HZ and JGa are mainly responsible for the design of the research. LW and YZ are mainly responsible for the evaluation and collection of the results. FM is responsible for the statistics of the data. YL is responsible for the guidance of the entire study. All authors contributed to the article and approved the submitted version.

## Funding

This work was supported by the Special Fund for Hospital Management Research (LC2020D02).

## Conflict of Interest

The authors declare that the research was conducted in the absence of any commercial or financial relationships that could be construed as a potential conflict of interest.

## Publisher's Note

All claims expressed in this article are solely those of the authors and do not necessarily represent those of their affiliated organizations, or those of the publisher, the editors and the reviewers. Any product that may be evaluated in this article, or claim that may be made by its manufacturer, is not guaranteed or endorsed by the publisher.
